# The Double-Edged Role of Enzyme Inhibition in Oral Anticancer Therapy

**DOI:** 10.3390/pharmaceutics18101251

**Published:** 2026-10-01

**Authors:** Abdullah A. Assiri

**Affiliations:** Department of Clinical Pharmacy, College of Pharmacy, King Khalid University, Abha 61421, Asir, Saudi Arabia; aalabdullah@kku.edu.sa

**Keywords:** enzyme inhibition, oral anticancer agents, cytochrome P450, enzyme induction, drug–drug interactions, pharmacogenomics, tyrosine kinase inhibitors

## Abstract

Oral anticancer therapy is built on the deliberate inhibition of enzymes that drive malignant growth, among them protein kinases, poly(ADP-ribose) polymerases, cyclin-dependent kinases 4 and 6, the PI3K/AKT/mTOR and BRAF/MEK cascades, histone deacetylases and the proteasome. The same molecules are processed by the enzymes that metabolize drugs, and several of them inhibit or induce those enzymes in turn. Enzyme inhibition therefore carries two faces, and the objective of this review is to examine them as a single pharmacological phenomenon rather than as two separate subjects. The argument proposed is that the two are causally linked, because the structural features allowing a molecule to occupy a catalytic site also determine which metabolizing enzymes recognize it. Three consequences organize the review. Agents sharing a therapeutic target can differ by more than an order of magnitude in their dependence on one metabolizing enzyme, meaning that interaction liability is a property of the molecule rather than of its class. The mechanism of inhibition, whether reversible, covalent or time-dependent, governs how long an interaction persists. Several oral anticancer agents are themselves inhibitors or inducers of drug-metabolizing enzymes, acting as perpetrators rather than victims. Each point is quantified for representative agents from regulatory pharmacology data.

## 1. Introduction

The pharmacological treatment of cancer has shifted decisively from cytotoxic chemotherapy toward agents that inhibit defined molecular enzymes. Between 2018 and 2023 alone, a large number of small-molecule kinase inhibitors entered clinical use, each engineered to block an enzyme that drives a specific malignant phenotype [1]. Many of these agents are taken orally and chronically in the outpatient setting, which fundamentally changes the interaction landscape compared with intermittent intravenous chemotherapy [2,3]. A growing share of anticancer treatment is now self-administered at home alongside whatever else a patient is taking, so systemic exposure comes to depend on the other prescriptions a patient carries, rather than on the controlled environment of an infusion suite.

The inhibition of enzymes in this setting is double-edged in a specific and mechanistically definable sense. When inhibition is directed at a cancer-driving enzyme such as an aberrant tyrosine kinase, a DNA-repair polymerase or a cell-cycle kinase, it is the intended therapeutic act. When the same molecular property falls instead on a drug-metabolizing enzyme such as CYP3A4, it becomes a pharmacokinetic perturbation that can raise exposure to toxic concentrations or, through enzyme induction, lower it below the threshold for efficacy [4,5]. These two phenomena are mechanistically distinct but clinically entangled, because the oral anticancer agent is at once an enzyme inhibitor and an enzyme substrate.

Oral anticancer agents are unusually vulnerable to the consequences of altered enzyme activity. Their oral bioavailability is frequently low and variable, and inter-individual exposure varies widely at fixed doses [2,3,6]. Many of them also have a narrow therapeutic window, which means that the separation between the exposure required for antitumor activity and the exposure at which dose-limiting toxicity appears is small. The practical consequence is that a change in exposure that would be inconsequential for a drug with a wide margin, such as the two-fold or three-fold rise that a strong CYP3A4 inhibitor routinely produces, is sufficient to move a patient from an effective to a toxic exposure or, in the case of enzyme induction, from an effective to a subtherapeutic one [7]. Ibrutinib illustrates the point at its extreme; because its absolute bioavailability is only 2.9%, almost the entire oral dose is removed by first-pass CYP3A extraction, and inhibiting that extraction with ketoconazole raises exposure twenty-four-fold. Chronic outpatient dosing maximizes the opportunity for polypharmacy, including acid-suppressing agents, antifungals and anticonvulsants [8,9,10], and cancer populations are older and more comorbid, with polypharmacy and potential drug–drug interactions both highly prevalent and independently associated with adverse treatment outcomes in older adults with advanced cancer [11].

**Figure 1:**
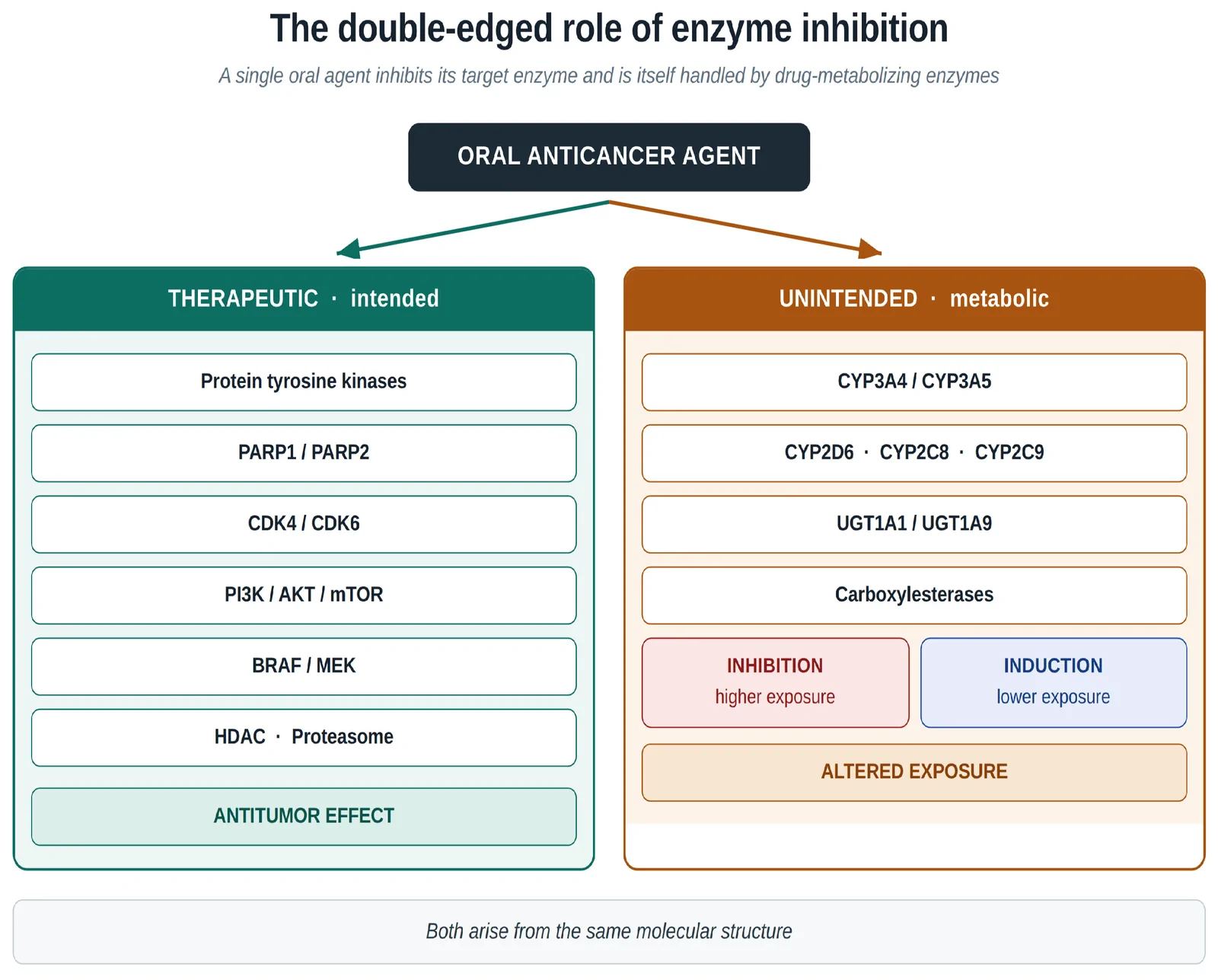
The double-edged role of enzyme inhibition in oral anticancer therapy. The left arm depicts the intended inhibition of cancer-related enzymes, including tyrosine kinases, PARP, CDK4/6, PI3K/AKT/mTOR, BRAF/MEK, HDAC and the proteasome, producing an antitumor effect. The right arm depicts the unintended modulation of drug-metabolizing enzymes, principally the cytochrome P450 isoforms and the uridine diphosphate glucuronosyltransferases, where inhibition raises exposure and toxicity, whereas induction lowers exposure and risks therapeutic failure. The dashed link indicates that a single oral agent participates in both processes simultaneously.

The objective of this review is to treat therapeutic enzyme inhibition and unintended enzyme inhibition or induction as shown in Figure 1 as one subject with a common molecular origin, and to show what follows from that for the safe use of oral anticancer agents. The organizing argument is that the structural features determining which catalytic site a molecule occupies also determine which metabolizing enzymes recognize it, meaning that therapeutic potency and interaction liability are generated together rather than independently. Three consequences follow and provide the structure of what follows. The first is that interaction liability resides in the individual molecule rather than in its therapeutic class, which is developed in Section 3. The second is that the mechanism of inhibition determines the duration and reversibility of an interaction, which is developed in Section 4 and Section 5. The third is that an anticancer agent may itself be the perpetrator of an interaction rather than its victim, which is developed in Section 6. The three are drawn together in Section 9, where each is stated with the evidence that supports it, its bearing on current practice and the research it calls for.

The scope of this review is deliberately confined to enzyme-mediated phenomena. The determinants of oral drug absorption, including food effects, gastric pH and acid-suppressive therapy, together with transporter-mediated disposition and the exposure–response relationships that motivate therapeutic drug monitoring, have recently been reviewed in detail elsewhere and are not revisited here [12]. Transporters are considered in this review only where transport and metabolism act on the same molecule and cannot be separated. What this review adds to that account is the enzymological dimension, namely the mechanism by which inhibition is achieved at both the therapeutic target and the metabolizing enzyme, and the consequences that follow from the mechanism itself.

## 2. Biochemical Basis of Oncogenic Enzyme Function

An account of why each of these enzymes is a therapeutic target is a necessary preliminary to any discussion of how they are inhibited. In every case the rationale rests on a specific biochemical lesion, namely an enzyme whose catalytic output has become constitutive, amplified or uniquely necessary to the malignant cell, such that removing that output damages the tumor more than the normal tissue around it.

Protein tyrosine kinases catalyze the transfer of the terminal phosphate of ATP to tyrosine residues on substrate proteins, and this phosphorylation acts as a molecular switch that recruits downstream signaling complexes [13]. Under physiological conditions the switch is transient and ligand-dependent, whereas oncogenic activation removes that dependence. In chronic myeloid leukemia, the t(9;22) translocation fuses BCR to ABL1, and the resulting fusion protein loses the autoinhibitory constraint imposed by myristoyl binding in native ABL1, becoming constitutively active and driving continuous proliferative signaling [14]. In non-small cell lung cancer, activating mutations in the EGFR kinase domain and chromosomal rearrangements that fuse ALK to a dimerization partner achieve the same end by different structural routes, since the kinase adopts its active conformation without ligand binding [15]. Because the transformed cell becomes dependent on the resulting signal, a phenomenon termed oncogene addiction, catalytic inhibition of the kinase produces apoptosis rather than mere growth arrest [16].

Poly(ADP-ribose) polymerases 1 and 2 detect single-strand DNA breaks and synthesize poly(ADP-ribose) chains that recruit the base-excision repair machinery, after which auto-modification releases the enzyme from DNA. Inhibiting this cycle has two distinct consequences that together explain the clinical behavior of the class. Unrepaired single-strand breaks are converted at the replication fork into double-strand breaks, which a cell with intact homologous recombination repairs but a cell with BRCA1 or BRCA2 loss cannot, and this is the basis of synthetic lethality. Separately, inhibitor binding stabilizes the enzyme on DNA rather than merely blocking catalysis, and these trapped PARP–DNA complexes are themselves cytotoxic lesions [17]. Trapping potency varies by orders of magnitude between agents and correlates with both antitumor activity and myelosuppression [18], which is why PARP inhibitors of comparable catalytic potency have markedly different hematologic profiles.

Cyclin-dependent kinases 4 and 6, acting in complexes with D-type cyclins, phosphorylate the retinoblastoma protein at the G1 restriction point. Hypophosphorylated retinoblastoma protein sequesters E2F transcription factors, and phosphorylation releases E2F and licenses transcription of the genes required for entry into S phase. In hormone-receptor-positive breast cancer, estrogen-driven cyclin D1 expression keeps this axis persistently active, meaning that inhibiting CDK4 and CDK6 restores retinoblastoma-mediated restraint and arrests cells in G1 [19].

The PI3K/AKT/mTOR cascade transduces growth-factor and nutrient signals, since class I PI3K phosphorylates phosphatidylinositol-4,5-bisphosphate to generate PIP3, which recruits AKT for activation, and activated AKT relieves inhibition of mTORC1, the serine/threonine kinase that couples nutrient availability to protein synthesis. Activating PIK3CA mutations and loss of PTEN render the pathway independent of upstream input [20]. In the MAPK cascade, BRAF V600 mutations produce a kinase that signals as a monomer independently of RAS and drives constitutive MEK and ERK activation, and inhibiting BRAF alone can paradoxically transactivate wild-type RAF dimers in cells with intact RAS signaling, which is the mechanistic reason that BRAF and MEK inhibitors are given together [21,22].

The two remaining target classes act on protein turnover rather than on signal transduction. Zinc-dependent histone deacetylases remove acetyl groups from lysine residues on histone tails, restoring the positive charge that tightens the interaction between histones and DNA and represses transcription, and their dysregulation in malignancy silences differentiation and tumor-suppressor programs [23]. The 20S proteasome degrades ubiquitin-tagged proteins through three distinct catalytic activities, of which the chymotrypsin-like activity of the beta-5 subunit is the principal drug target. Malignant plasma cells synthesize immunoglobulin at extreme rates, carry a correspondingly heavy load of misfolded protein and are therefore dependent on proteasomal disposal, meaning that inhibition precipitates terminal endoplasmic-reticulum stress and apoptosis. This dependence explains why proteasome inhibition is effective in myeloma and largely inactive elsewhere [24].

A theme running through all of these mechanisms is that the therapeutic target is a catalytic site, and that the molecule designed to occupy it must combine high affinity with sufficient selectivity to spare the many structurally related enzymes in the same family. As Section 4 develops in detail, the chemical solutions to that problem simultaneously determine how the molecule is metabolized and cleared, which is the reason this review treats target pharmacology and interaction liability as one subject rather than two.

## 3. Therapeutic Enzyme Inhibition and Its Metabolic Counterpart

Therapeutic enzyme inhibition spans several mechanistic classes, running from the protein and lipid kinases that transduce growth signals to the DNA-repair, epigenetic and protein-degradation enzymes that sustain the malignant phenotype [1,25]. Table 1 consolidates these targets, their representative oral agents and the mechanism by which each is inhibited. The clinically important observation, and the first of the three consequences set out in the introduction, is that agents which share a target do not share an interaction profile, because the enzyme that clears a molecule is determined by its chemistry rather than by its pharmacological class.

### 3.1. Protein Tyrosine Kinases

Tyrosine kinase inhibitors are the largest and most heterogeneous class of oral targeted agents [1,25]. Most are ATP-competitive inhibitors that occupy the catalytic cleft, but they differ in binding mode, in the kinase conformation they recognize and in the breadth of off-target activity, and these structural differences have been systematically classified from crystallographic structures of drug–enzyme complexes [56]. Their oral pharmacokinetics are notoriously variable, since bioavailability is frequently incomplete and inter-patient exposure can differ several-fold at an identical dose [2,3,6]. Combined with chronic daily dosing, these features make metabolism-related interactions a defining concern for the class and have motivated a substantial literature on the pitfalls of prescribing [4,57,58].

#### 3.1.1. BCR-ABL1 Inhibitors

In chronic myeloid leukemia, BCR-ABL1 inhibitors block the constitutively active fusion kinase that drives the disease [26,59]. Resistance arising from kinase-domain mutations, most notably the T315I gatekeeper substitution, motivated successive generations of agents, and asciminib is mechanistically distinct because it binds the myristoyl pocket rather than the ATP site and thereby restores the autoinhibitory conformation that the fusion protein has lost [27,60]. Because exposure correlates with both molecular response and toxicity and varies widely between patients, including pediatric populations, therapeutic drug monitoring and germline pharmacogenetics have been examined across imatinib and its successors [28,61,62,63,64,65]. The enzymological consequence of these structural differences is a twenty-fold range of CYP3A4 dependence across agents that share a single target.

#### 3.1.2. EGFR, ALK and MET Inhibitors

In non-small cell lung cancer, EGFR inhibitors block signaling from activating kinase-domain mutations, ALK inhibitors target ALK rearrangements across successive generations of increasing potency and central nervous system penetration, and MET inhibitors address exon 14 skipping alterations [29,30,31,66]. Both germline and somatic variation influence efficacy and toxicity, so molecular profiling is increasingly integrated with pharmacology in treatment selection [67,68,69]. Class-specific adverse effects require proactive management, since crizotinib carries cardiac and visual effects, ceritinib pronounced gastrointestinal toxicity and lorlatinib a distinctive metabolic and neurocognitive profile [70,71,72,73,74]. Several of these agents undergo metabolic activation to reactive intermediates, which links their structure both to idiosyncratic toxicity and to time-dependent inhibition of the enzymes that generate those intermediates [75,76].

#### 3.1.3. VEGFR Inhibitors

Multitargeted VEGFR inhibitors inhibit angiogenic kinases and share a class hypertension signal that reflects on-target blockade of the VEGF pathway rather than an off-target liability [32,33,34,77,78,79]. Broad kinase coverage contributes both to activity across tumor types and to a wide adverse-event spectrum, so managing toxicity is integral to preserving the dose intensity on which efficacy depends [80,81]. The enzymology of this class is distinctive because clearance is divided between CYP3A4 oxidation and UGT1A9 glucuronidation, and the parallel route can buffer the loss of oxidative clearance to the point that a strong CYP3A4 inhibitor produces no change in exposure at all [82]. Exposure variability within the class has prompted investigation of therapeutic drug monitoring, most actively in renal cell carcinoma [83].

#### 3.1.4. Bruton Tyrosine Kinase Inhibitors

Bruton tyrosine kinase inhibitors used in B-cell malignancies are covalent agents that bind a cysteine residue in the active site, achieving prolonged target engagement that persists after the drug itself has been cleared [35,36]. Their exposure is highly dependent on CYP3A, so interaction management and dose modification feature prominently in guidance for the class [37,84]. This class demonstrates the argument of the review most directly, because the same structural decisions separate its members both therapeutically and metabolically. Ibrutinib inhibits EGFR at 5.3 nM, ITK at 4.9 nM and TEC at 10 nM, which is close to its potency against its intended target, whereas acalabrutinib exceeds 1000 nM against EGFR and ITK, and that difference in off-target coverage accounts for the difference in toxicity between them. Independently of it, the low first-pass survival of ibrutinib means that almost the entire oral dose is removed by intestinal and hepatic CYP3A extraction, meaning that inhibiting the enzyme raises exposure twenty-four-fold against approximately four-fold for zanubrutinib. The practical result is that ibrutinib is the wrong member of its class for a patient who requires long-term azole antifungal prophylaxis.

#### 3.1.5. Other Kinase Families

Several further oral agents extend the same logic to additional kinase families. The JAK inhibitor ruxolitinib, used in myeloproliferative neoplasms, is itself a CYP3A4 substrate, and ketoconazole raises its exposure by 91% while rifampin lowers it by 61% [85,86]. In acute myeloid leukemia, the FLT3 inhibitor quizartinib carries its own interaction considerations against a background of frequent antifungal co-therapy, and ketoconazole raises its exposure by 94%, with the labeled dose reduction driven by QT prolongation rather than by myelosuppression [87,88]. The CSF1R inhibitor pexidartinib extended oral targeted therapy into non-malignant proliferative disease and is cleared by both CYP3A4 oxidation and UGT1A4 glucuronidation, meaning that inhibitors of either enzyme raise its exposure and hepatotoxicity monitoring is required [89].

### 3.2. PARP Enzymes

PARP inhibitors exploit synthetic lethality in tumors with homologous-recombination deficiency and additionally trap the enzyme on DNA, a mechanism that contributes both to antitumor activity and to the hematologic toxicity that characterizes the class [17,38,39,90]. Comparative studies establish talazoparib as the most potent trapper, although published rankings for the intermediate agents are discordant [18]. Combination strategies that pair PARP inhibitors with other targeted agents raise additional questions of overlapping toxicity and pharmacokinetic interaction [91]. Because the relationship between exposure and both response and myelosuppression is relatively narrow, validated bioanalytical methods have been developed and there is growing interest in model-informed dosing for the class [40,92,93,94,95].

This class makes the argument of the review from the opposite direction to the BTK agents, because four drugs that share a target and a trapping mechanism are cleared by four different routes. Olaparib is the only member that depends substantially on CYP3A, and it is correspondingly the only one for which labeling mandates a CYP3A-based dose reduction. Rucaparib is metabolized principally by CYP2D6 and requires no such adjustment, while niraparib bypasses cytochrome P450 almost entirely because it is hydrolyzed by carboxylesterases before glucuronidation. Talazoparib undergoes minimal metabolism of any kind and is excreted largely unchanged, so the changes in its exposure observed with itraconazole and rifampin are transporter effects rather than enzyme effects, and reporting them as CYP3A findings is a factual error with practical consequences.

### 3.3. CDK4 and CDK6

CDK4/6 inhibitors block phosphorylation of the retinoblastoma protein to arrest cell-cycle progression and have become central to the treatment of hormone-receptor-positive, HER2-negative breast cancer [41,96,97]. The three approved agents are not interchangeable, since they differ in relative potency against CDK4 compared with CDK6 and in toxicity profile, and all three are CYP3A4 substrates, so a review of concomitant medication is integral to their safe use [42,98]. Characteristic adverse events shape monitoring, with neutropenia across the class, QT prolongation with ribociclib, diarrhea with abemaciclib, and uncommon but relevant interstitial lung disease [43,99]. As these agents move into the adjuvant setting in older and more comorbid patients, structured interaction and comorbidity management becomes correspondingly more important [100,101].

The enzymological point in this class runs counter to the usual emphasis, because the interaction magnitudes are modest while the pharmacodynamic differences are large. Induction rather than inhibition is the dominant hazard, since rifampin reduces exposure by between 85% and 89% for all three agents and strong inducers are therefore contraindicated, whereas a strong inhibitor produces changes of between two-fold and three-fold. What drives selection between the three is the potency ratio against CDK4 compared with CDK6 and the toxicity that follows from it, with the concentration-dependent QT signal of ribociclib making its dose reduction with a strong inhibitor a safety requirement rather than a tolerability measure [43].

### 3.4. PI3K, AKT and mTOR Pathway Enzymes

Inhibitors of the phosphatidylinositol 3-kinase, AKT and mTOR cascade act on lipid and serine-threonine kinases that integrate growth, metabolic and survival signaling, a pathway whose role in several malignancies has made it an enduring focus of drug development [102]. The class includes the PI3K-delta inhibitor idelalisib, used in B-cell malignancies, and the mTOR inhibitors everolimus and sirolimus, whose use in oncology is informed by an unusually deep body of transplant pharmacology [44,45,103]. The mTOR inhibitors are instructive because they are the most CYP3A4-sensitive agents considered in this review, and ketoconazole raises everolimus exposure fifteen-fold and sirolimus exposure almost eleven-fold, which is why their concentrations are actively controlled rather than merely monitored [46]. Combined blockade of this pathway together with angiogenic signaling has also been explored, adding further interactions to anticipate [103].

### 3.5. BRAF and MEK

In BRAF-mutant melanoma, BRAF inhibitors such as vemurafenib and dabrafenib are routinely combined with MEK inhibitors to deepen and prolong responses and to suppress the paradoxical activation of the MAPK pathway that BRAF inhibition alone can provoke in cells with wild-type BRAF [47,48]. The two BRAF agents differ in pharmacokinetics, and the capacity of dabrafenib to induce its own metabolism is directly relevant to dosing and to the prediction of interactions [48]. MEK inhibitors are allosteric agents that bind outside the ATP site and carry their own toxicities, and the cutaneous adverse events of pathway-directed therapy are a recognizable signature of the class [49,104]. The combination has been extended beyond melanoma, including to pediatric glioma, where efficacy must be balanced against a distinct toxicity spectrum in a vulnerable population [105].

### 3.6. Histone Deacetylases

Histone deacetylase inhibitors inhibit zinc-dependent deacetylases, restoring physiological histone acetylation, reprogramming gene expression and exerting immunomodulatory effects that are increasingly recognized as part of their mechanism [50,106,107]. Two points require care in this class. Only vorinostat and panobinostat are administered orally, whereas belinostat and romidepsin are given intravenously, and the clearance route rather than the class determines interaction risk, since vorinostat is cleared by glucuronidation and hydrolysis with negligible cytochrome P450 involvement, while panobinostat depends on CYP3A for approximately 40% of its hepatic elimination. Belinostat remains the clearest illustration in oncology of an agent whose clearance is dominated by UGT1A1 rather than by cytochrome P450, and its labeled dose reduction is genotype-based, although no clinical study of UGT1A1-inhibiting comedication was conducted and the quantitative genotype estimate in the regulatory record rests on simulation [51,108,109,110]. Beyond their direct antitumor action, these agents modulate the expression of efflux transporters, a pharmacodynamic effect that feeds back into pharmacokinetics [52], and they are frequently studied alongside platinum agents, kinase inhibitors and hypomethylating agents, each pairing carrying its own interaction profile [111,112,113].

### 3.7. Proteasome-Associated Targets

Proteasome inhibitors block the catalytic activities of the 20S proteasome, exploiting the dependence of malignant plasma cells on protein turnover and thereby anchoring the treatment of multiple myeloma [53]. Bortezomib and carfilzomib are administered parenterally, whereas ixazomib is the first oral proteasome inhibitor, a development that moved this mechanism into chronic outpatient therapy and into the same interaction landscape as the oral kinase inhibitors [54]. The agents differ in the reversibility of proteasome binding, in off-target activity and in pharmacokinetics, and these differences translate into distinct efficacy and tolerability profiles [55]. Managing the characteristic toxicities of myeloma therapy, and peripheral neuropathy in particular, is integral to their clinical use and a frequent driver of dose adjustment [114,115]. The enzymology of ixazomib is unusual because no single enzyme dominates its clearance, meaning that a strong inhibitor produces no clinically meaningful change while rifampin reduces exposure by 74%, and the actionable instruction therefore concerns inducers rather than inhibitors.

Table 2 collects representative agents from each of these classes and sets out, for each of them, the parameters on which the comparison rests. They are confined to enzymology, namely the potency and mechanism of inhibition at the therapeutic target, the enzyme that dominates clearance of the molecule, the measured change in exposure produced by a strong inhibitor and by a strong inducer of that enzyme, and the dose modification that product labeling mandates as a result.

The extremes of the whole dataset are worth stating explicitly because they define the range a prescriber must consider. Everolimus is the most enzyme-dependent agent in this review, and ketoconazole raises its exposure fifteen-fold. At the other extreme, alectinib shows no clinically meaningful change with either a strong inhibitor or a strong inducer, and vorinostat is cleared by glucuronidation and hydrolysis with negligible cytochrome P450 involvement. Between these poles lies a continuous distribution rather than a set of classes, and an agent’s position on it is not predictable from its therapeutic target.

## 4. Molecular Basis of Inhibitor Selectivity and Its Metabolic Consequence

Inhibitor selectivity is the central design problem of enzyme-targeted oncology, and it propagates into potency, off-target liability, resistance behavior and ultimately the interaction profile of the finished drug. Most kinase inhibitors are ATP-competitive and must achieve selectivity despite binding a catalytic cleft that is structurally conserved across hundreds of human kinases. They do so by exploiting differences in the conformation the kinase adopts, principally the DFG-in and DFG-out states and the position of the alpha-C helix, and in the size of the gatekeeper residue that guards a hydrophobic back pocket. The resulting binding modes have been formally classified from crystallographic structures of drug–enzyme complexes into type I, type I-and-a-half, type II and the allosteric type III and IV categories [56]. The structural features that confer high affinity for the target frequently dictate which metabolic enzymes recognize and process the molecule, meaning that inhibitor design determines metabolic fate and susceptibility to interaction at the same time as it determines potency.

**Figure 2:**
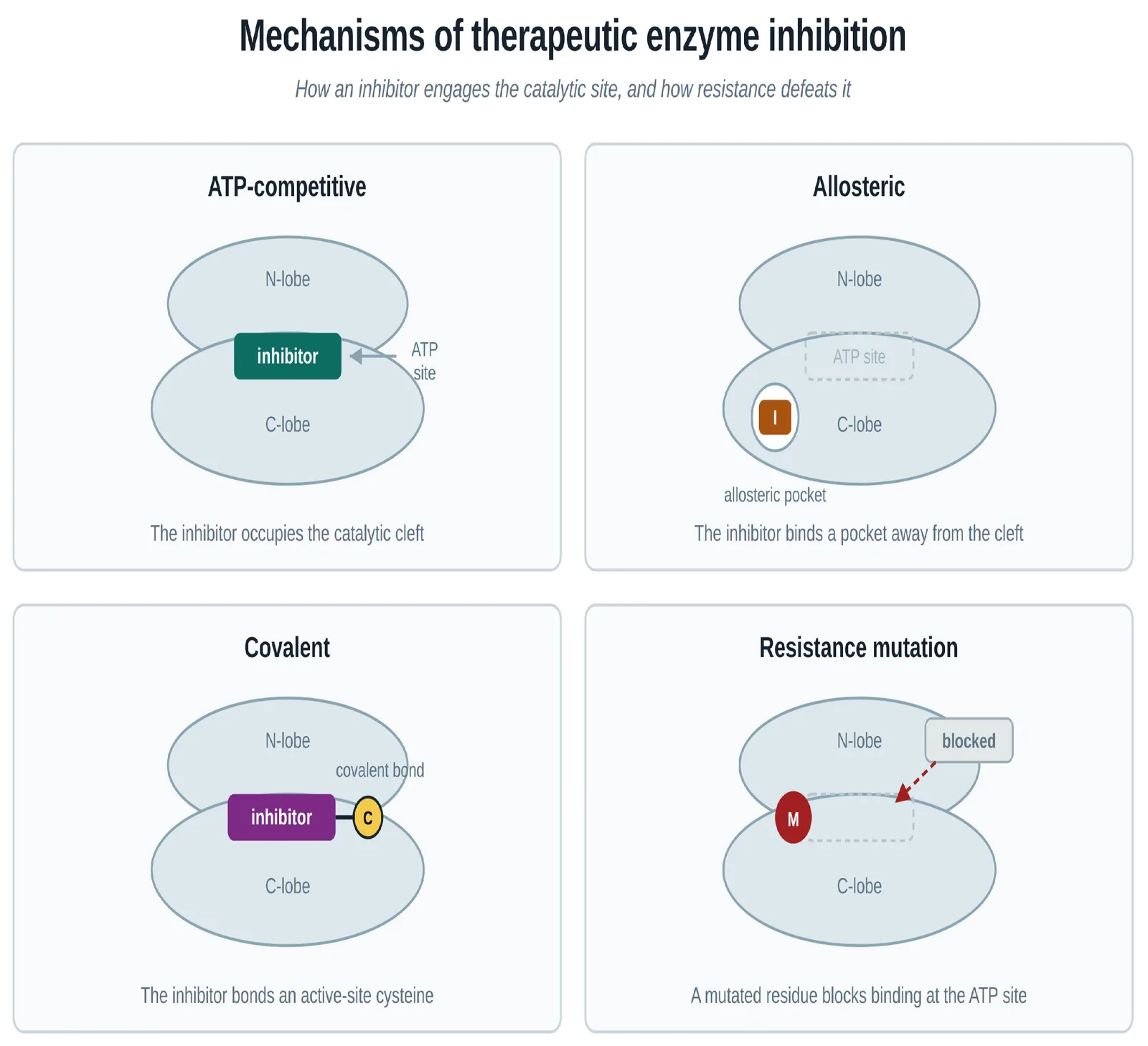
Molecular mechanisms of therapeutic enzyme inhibition. ATP-competitive inhibitors occupy the catalytic cleft, allosteric inhibitors bind a distinct pocket such as the myristoyl pocket engaged by asciminib, covalent inhibitors form a bond with an active-site cysteine as in the BTK inhibitors, and a resistance mutation introduces a residue that sterically blocks ATP-competitive binding while frequently sparing allosteric sites. In the panels, I denotes the inhibitor, C the active-site cysteine to which a covalent inhibitor binds, and M the mutated residue.

Several strategies beyond classical ATP competition are clinically relevant and are represented in Figure 2. Allosteric inhibition binds a site distinct from the catalytic center, and the binding of asciminib to the myristoyl pocket of ABL1 is a clear oncology example, as well as a route around resistance mutations at the ATP site [27]. Covalent inhibition forms a bond, usually with an active-site cysteine, which confers prolonged target engagement that persists after the drug itself has been cleared, as comparative analysis of approved covalent and reversible inhibitors has shown for the BTK agents [116]. Reversible non-covalent binding, by contrast, ties the pharmacodynamic effect more tightly to the prevailing plasma concentration.

Medicinal-chemistry optimization tunes these properties through pharmacophore features and scaffold choice. Heterocyclic scaffolds such as pyrimidines recur across inhibitor classes, and small strained rings such as oxetanes have been used to improve metabolic stability and physicochemical behavior [117,118]. Polypharmacology can also be designed intentionally, as in dual BRD4 and kinase inhibitors [119]. Because the chemical features that confer target affinity also shape metabolic susceptibility, and because many tyrosine kinase inhibitors undergo metabolic activation to reactive intermediates [75], selectivity, resistance and interaction risk are coupled consequences of the same molecular choices. The BTK agents in Table 2 are the clearest empirical demonstration of that coupling, since the kinase-selectivity difference between ibrutinib and acalabrutinib explains their divergent toxicity while the first-pass extraction difference between them explains their divergent interaction magnitude, and both differences follow from the same structural decisions.

## 5. Unintended Inhibition and Induction of Drug-Metabolizing Enzymes

The same enzymatic machinery that clears oral anticancer agents is also the locus of their most important pharmacokinetic interactions. Modulation of a drug-metabolizing enzyme by a co-administered drug alters systemic exposure in ways that are frequently predictable once the responsible pathway is known [1,120]. The task facing a prescriber is therefore less a matter of memorizing individual interaction pairs than of identifying the enzyme that governs a given agent and anticipating what a strong inhibitor or inducer of that enzyme will do to its exposure.

Cytochrome P450 3A4, together with the polymorphic CYP3A5, dominates the metabolism of most oral targeted agents and is the single most important interaction node in oral oncology [4,5]. Strong inhibitors of this enzyme, principally the azole antifungals, several macrolide antibiotics and some antiretrovirals, can raise the exposure of a sensitive substrate several-fold, while strong inducers such as rifampin and the enzyme-inducing antiepileptic drugs can lower it far enough to threaten efficacy [9,10]. The frequent co-prescription of azole antifungals during the cytopenic phases of treatment makes the combination of an antifungal with a targeted agent one of the most clinically relevant illustrations of this hazard [9]. The magnitude of any given effect scales with the completeness of an agent’s dependence on the enzyme in question, a principle borne out by systematic analyses of interactions among recently approved drugs [121] and quantified agent by agent in Table 2.

Other cytochrome P450 isoforms contribute in specific contexts and are easily overlooked when screening concentrates on CYP3A4 alone. CYP2D6 governs the conversion of tamoxifen to its active metabolite endoxifen, and CYP2C8 is the dominant route for enzalutamide and a major contributor for dabrafenib, which is why gemfibrozil rather than an azole is the perpetrator that forces a dose reduction for those two agents. CYP2C9, CYP2C19 and CYP1A2 contribute to the metabolism of selected agents, but agent-specific data in oncology are more limited than for CYP3A4, so their interactions are often inferred from substrate status rather than measured [122]. Broader roles for cytochrome P450 enzymes in cancer biology, including estrogen biosynthesis and xenobiotic handling, have also been described [123,124,125].

The way in which gemfibrozil inhibits CYP2C8 deserves to be stated precisely, because it bears on the mechanistic argument of this review. Gemfibrozil itself is only a weak direct inhibitor of the enzyme, and the clinically important effect is produced by its 1-O-β-glucuronide, which CYP2C8 converts to a species that inactivates the enzyme irreversibly, the concentration required for half-maximal inhibition falling from 24 μM to 1.8 μM after preincubation with human liver microsomes and NADPH [126]. Clopidogrel behaves in the same way, since its acyl-β-D-glucuronide rather than the parent drug is a strong time-dependent inhibitor of CYP2C8 [127]. A phase II conjugate is therefore the perpetrator in both cases, which screening confined to the parent molecule will miss. The consequences were established outside oncology, when gemfibrozil raised plasma cerivastatin exposure to more than five times its control value by blocking the CYP2C8-dependent step in its metabolism [128], and the combination was implicated in the fatal rhabdomyolysis that led to the withdrawal of cerivastatin from the world market in 2001 [129]. Because the inactivation is mechanism-based rather than competitive, its offset is governed by resynthesis of the enzyme rather than by elimination of the perpetrator.

Glucuronidation by the uridine diphosphate glucuronosyltransferases is the most clinically important non-cytochrome pathway in this setting, and Table 2 shows how frequently it determines whether an agent is vulnerable to cytochrome P450 modulation at all. Polymorphisms in UGT1A1 strongly influence the toxicity of the active irinotecan metabolite SN-38 and of the topoisomerase-inhibitor payload delivered by the antibody–drug conjugate sacituzumab govitecan, and the same enzyme governs the clearance of belinostat [51,110,130,131,132,133,134]. Glucuronidation also shapes the disposition of sorafenib and regorafenib, where a parallel UGT1A9 route buffers the inhibition of CYP3A4 to the point that ketoconazole does not alter sorafenib exposure at all. An agent’s vulnerability is therefore defined by the enzyme that dominates its clearance and not by its therapeutic class.

Transport and metabolism act on many of the same molecules and cannot always be separated, which is the one respect in which this review must look beyond the enzymes themselves. P-glycoprotein and BCRP handle many of the same substrates as CYP3A4, and their inhibition or induction can act in concert with a metabolic interaction [135,136,137]. For a small number of agents, transport is not merely additive but dominant, meaning that the dose modifications mandated for afatinib are keyed to P-glycoprotein rather than to any enzyme, and the apparent interactions of talazoparib are transporter effects in a drug that undergoes almost no metabolism at all. Many kinase inhibitors are both substrates and inhibitors of these proteins [138,139], and because transporter expression can itself be induced or suppressed, the contribution is dynamic rather than fixed, while continuing medicinal chemistry work is defining how selectively these proteins can be modulated [140]. Where transport rather than metabolism governs an interaction, screening confined to cytochrome P450 pathways will miss it.

**Figure 3:**
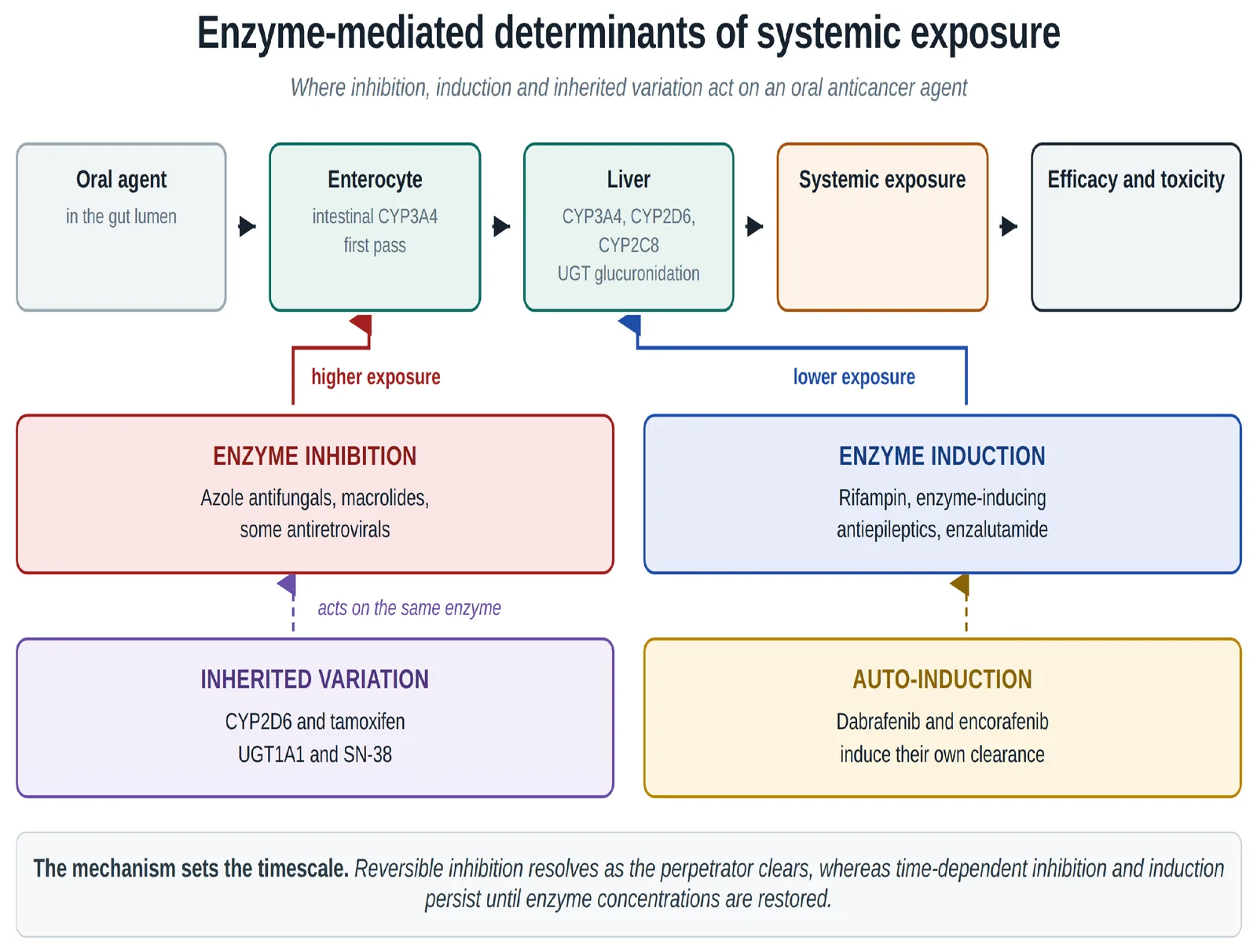
Enzyme-mediated determinants of systemic exposure for an oral anticancer agent. The agent encounters intestinal CYP3A4 on first pass and then hepatic cytochrome P450 and glucuronosyltransferase activity before reaching the systemic circulation. Inhibition at either site raises exposure and toxicity, whereas induction lowers exposure and risks therapeutic failure. Inherited variation in the metabolizing enzyme acts on the same node as a co-administered inhibitor, and an agent that induces its own clearance acts on that node itself.

A mechanistic distinction that is frequently omitted from interaction screening matters a great deal for risk assessment, and it constitutes the second of the three consequences set out in the introduction. Reversible inhibition resolves as the perpetrator is cleared, meaning that the interaction lasts the duration of the perpetrator’s own elimination. Time-dependent inhibition, also termed mechanism-based inhibition, inactivates the enzyme itself and produces longer-lasting and sometimes cumulative effects that persist until a new enzyme is synthesized, and the metabolic activation of several tyrosine kinase inhibitors to reactive species is one route to such inhibition [1,75]. Enzyme induction operates on a third timescale entirely, since it requires transcriptional upregulation and new protein synthesis to develop and an equivalent period to decay. The anatomical site of the effect is also decisive, because intestinal CYP3A4 acts on first pass, and inhibition there can disproportionately raise the bioavailability of a drug with low first-pass survival, independently of any hepatic effect [3,141]. Ibrutinib, with an absolute bioavailability of 2.9% and a twenty-four-fold interaction with ketoconazole, is the arithmetic demonstration of that principle. Table 3 organizes these mechanistic categories and Figure 3 maps them onto the path an oral agent follows from gut to systemic exposure.

## 6. Oral Anticancer Agents as Perpetrators of Enzyme-Mediated Interactions

The third consequence of treating enzyme inhibition as a single phenomenon is that the direction of an interaction cannot be assumed. An oral anticancer agent designed to inhibit one enzyme may inhibit or induce another, and when it does so it becomes the perpetrator of an interaction rather than its victim. This possibility is easily missed, because interaction screening in oncology is habitually organized around the anticancer agent as the drug at risk. Table 4 collects the agents for which a perpetrator effect has been measured against a probe substrate.

A small number of oral agents sit at the top of the interaction-risk hierarchy because they combine heavy dependence on a single enzyme with a narrow margin between effective and toxic exposure and frequent co-administration with strong perpetrators [4,5]. Venetoclax is the clearest example: since ketoconazole raises its exposure by 540% and ritonavir by 690%, and because the risk of tumor lysis syndrome is concentrated in the dose ramp-up phase, strong CYP3A inhibitors are contraindicated during initiation rather than merely requiring dose reduction [143,144]. Ibrutinib is comparable in magnitude and is managed by named agent-specific reduced doses [84,145]. Among the BCR-ABL1 inhibitors, dasatinib, nilotinib and bosutinib are CYP3A4 substrates whose labeled responses to an identical perpetrator differ from numeric reduction to outright prohibition [61,146,147], and the VEGFR-directed agents used in renal and thyroid cancer require parallel attention to their own enzymatic clearance and to the class hypertension signal [32,33,148].

Auto-induction is the most instructive case, because the agent acts as a perpetrator upon itself. Dabrafenib induces CYP3A4 and CYP2B6 through activation of the pregnane X receptor, an effect confirmed by a two-fold rise in the urinary ratio of 6-beta-hydroxycortisol to cortisol. Its own exposure on day 15 is 47% lower than on day 1, its apparent clearance approximately doubles, and it reduces midazolam exposure by 74%. Encorafenib induces its own metabolism as well, with the practical consequence that the efficacy of hormonal contraception is reduced during treatment. The clinical implication of auto-induction is that single-dose pharmacokinetic data systematically overestimate steady-state exposure, and that a co-medication started at the beginning of treatment may become subtherapeutic without any change in its own dose.

Idelalisib illustrates the same principle in the opposite direction and at greater magnitude. This agent is a therapeutic inhibitor of the delta isoform of PI3K, and it is simultaneously a strong inhibitor of CYP3A, raising midazolam exposure 5.4-fold. An anticancer drug that inhibits CYP3A more strongly than several of the antifungals routinely discussed as perpetrators is a clear demonstration that the roles of victim and perpetrator are not fixed by drug category. Several other agents inhibit metabolizing enzymes at clinically relevant concentrations, including nilotinib and rucaparib against UGT1A1 and asciminib against CYP3A4 and CYP2C9, and vemurafenib inhibits a broad range of cytochrome P450 isoforms. The androgen-axis antagonists abiraterone and enzalutamide are the best-characterized perpetrators in oral oncology, acting respectively through CYP2D6 inhibition and strong CYP3A4 induction, and their behavior has been described in detail elsewhere [12,142,149,150].

**Table 4:** Oral anticancer agents that act as perpetrators of enzyme-mediated interactions, including those that induce their own clearance.

Agent	Enzyme Affected	Direction and Mechanism	Measured Effect on a Probe or Co-Administered Substrate	Practical Consequence
Enzalutamide	CYP3A4, CYP2C9, CYP2C19	Strong induction	Midazolam exposure reduced by 86%, S-warfarin by 56%, omeprazole by 72%	Slow onset and offset, with a terminal half-life of 5.8 days, so a washout is required before starting a sensitive CYP3A substrate [142]
Abiraterone acetate	CYP2D6, CYP2C8	Reversible inhibition	Dextromethorphan exposure raised 2.9-fold; pioglitazone raised 46%	Review of concomitant CYP2D6 substrates is required, and dose reduction of the substrate may be needed [149,150]
Idelalisib	CYP3A	Strong inhibition	Midazolam exposure raised 5.4-fold	Sensitive CYP3A substrates should be avoided during treatment [44]
Dabrafenib	CYP3A4, CYP2B6	Auto-induction through pregnane X receptor activation	Own exposure 47% lower at day 15 than day 1; midazolam exposure reduced 74%	Single-dose data overestimate steady-state exposure, and co-medications may drift subtherapeutic [48]
Encorafenib	CYP3A4	Auto-induction	Steady-state exposure lower than after the first dose; magnitude not quantified	Hormonal contraceptive efficacy is reduced, and non-hormonal contraception is required [8]
Nilotinib	UGT1A1	Reversible inhibition	Inhibition demonstrated in vitro at clinically relevant concentrations	Caution with substrates cleared by UGT1A1, including irinotecan payloads [28]
Rucaparib	UGT1A1, CYP2C9, CYP2C19	Reversible inhibition	Inhibition of multiple enzymes and transporters at clinical exposures	Labeling directs reduction in the co-administered substrate rather than of rucaparib [40,92]
Asciminib	CYP3A4, CYP2C9	Reversible inhibition	Inhibition demonstrated at clinically relevant concentrations	Review of concomitant substrates of both enzymes is required [27]
Vemurafenib	CYP1A2, CYP2C9, CYP3A4 and others	Reversible inhibition of multiple isoforms	Broad inhibition reported in the regulatory record without a single quantified probe result	Interaction potential is broad but poorly quantified, and monitoring is the practical response [47]

## 7. Inherited Variation in Drug-Metabolizing Enzymes

Inherited variation in the genes encoding drug-metabolizing enzymes produces the same pharmacokinetic consequence as a drug that inhibits those enzymes, and the two mechanisms are additive. Pharmacogenomics, in the sense used here and in regulatory guidance, is the study of how such inherited variation alters an individual’s response to a medicine, and it is distinct from the study of how a drug interacts physically with nucleic acids. Two gene–drug pairs in oncology are actionable, and both concern enzymes central to this review.

The relationship between CYP2D6 and tamoxifen is the first of these pairs. Tamoxifen is itself a weak antiestrogen, and its activity depends on conversion by way of N-desmethyltamoxifen to endoxifen, which has roughly one hundred times greater affinity for the estrogen receptor than the parent drug. CYP2D6 catalyzes that conversion, so reduced-function genotypes yield lower endoxifen concentrations, and in a genotype-guided dose-escalation study the median endoxifen concentration at the standard 20 mg daily dose was 8.0 nM in poor metabolizers, 17.8 nM in intermediate metabolizers and 33.7 nM in normal metabolizers, with genotype explaining between 34% and 52% of the variance [151,152,153]. The same phenotype can be produced pharmacologically, since among genotypically normal metabolizers the mean steady-state endoxifen concentration was 31.4 ng/mL without a CYP2D6 inhibitor and 8.8 ng/mL in those taking a potent inhibitor such as paroxetine or fluoxetine. That comparison is the point at which inherited variation and drug interaction become the same clinical problem, and consortium guidance accordingly recommends avoiding moderate and strong CYP2D6 inhibitors in normal and ultrarapid metabolizers and considering an aromatase inhibitor in poor and intermediate metabolizers [153].

The relationship between UGT1A1 and irinotecan is the second pair. SN-38, the active metabolite of irinotecan, is detoxified by UGT1A1-mediated glucuronidation, and the UGT1A1*28 allele carries an additional TA repeat in the promoter that reduces transcription by approximately 35% with one allele and 70% with two. Homozygosity increases systemic exposure to SN-38 by between 18% and 159% while reducing its metabolic clearance by 61% [130,131,132,133,134]. The clinical consequence is dose-dependent, since grade 4 neutropenia occurred in 50% of *28/*28 patients and in none of the *1/*1 patients at a single-agent dose of 350 mg/m^2^, and a meta-analysis reported odds ratios for severe neutropenia rising from 1.80 at low dose to 27.8 at high dose [132,134]. Allele frequency varies markedly by ancestry, ranging from 0.42 to 0.45 in African American populations and from 0.09 to 0.16 in East Asian populations, the latter making concurrent genotyping of the *6 allele necessary. Actionable guidance at present comes from the Dutch Pharmacogenetics Working Group, which recommends starting poor metabolizers at 70% of the normal dose, together with product labeling, since a consortium guideline for this pair has been identified as a priority but has not yet been issued [154,155].

## 8. Applying Enzyme Mechanism to Prescribing Decisions

**Figure 4:**
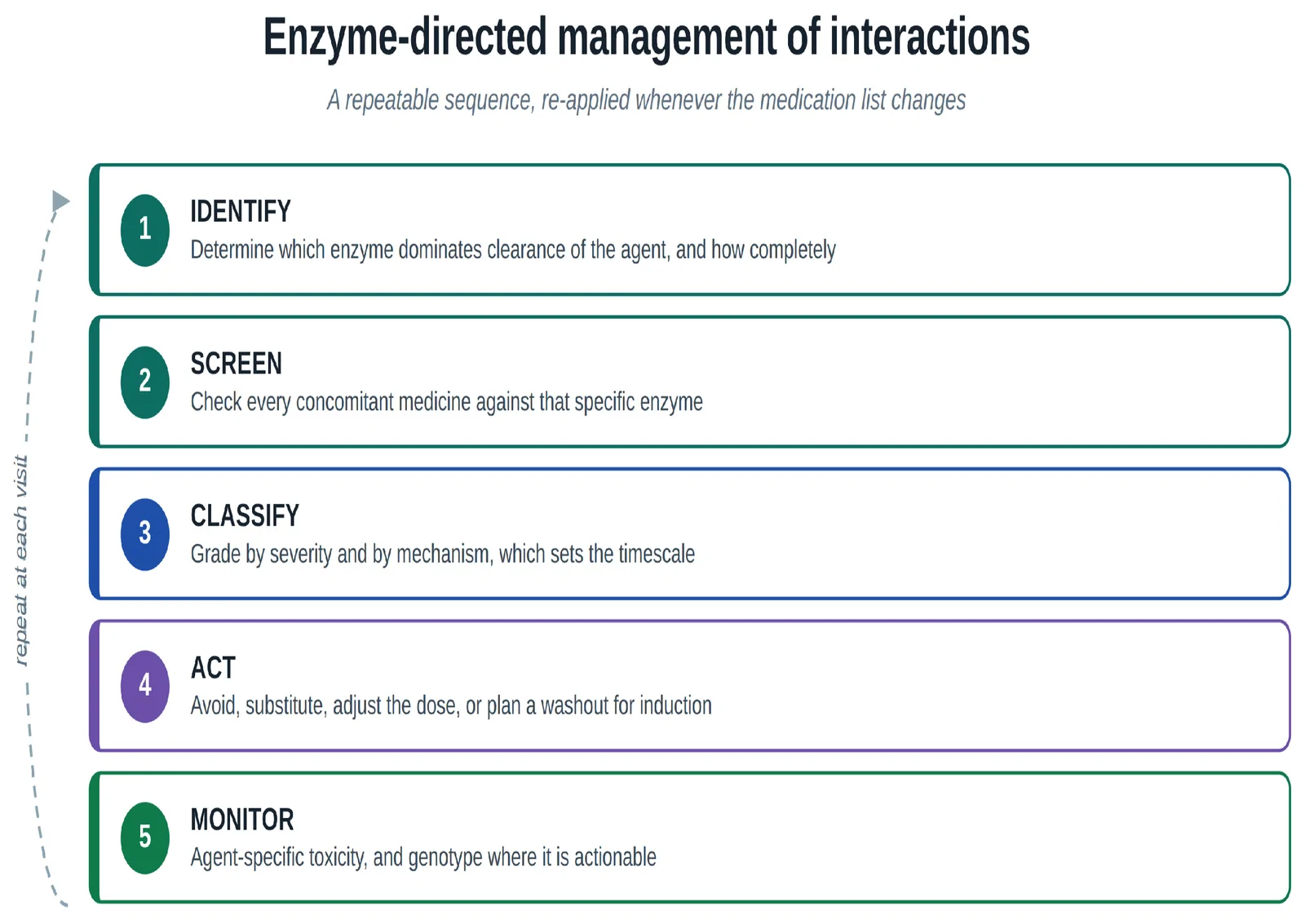
Enzyme-directed workflow for managing interactions in oral anticancer therapy. The sequence runs from identification of the enzyme that dominates clearance of the agent, through screening of concomitant medicines against that enzyme, classification of each interaction by mechanism and severity, action, and monitoring. The dashed loop indicates that the sequence is repeated whenever the medication list changes.

The management of enzyme-mediated interactions in oral oncology is best understood as a structured and repeatable process applied at every clinical encounter as the medication list evolves, rather than as a single intervention. Figure 4 sets out that sequence, and the text below states only what the figure cannot, namely the enzymological judgment each step requires.

The characterization step asks not which class the anticancer agent belongs to but which enzyme dominates its clearance and how completely, since Table 2 shows that the answer differs within every class examined. Screening then converts an unwieldy list of theoretical interactions into a short and ranked set by asking, for each concomitant drug, whether it modulates that specific enzyme [4,5,57]. The decision step selects among avoidance, substitution, dose adjustment and intensified monitoring, and the agent-level data show how widely the mandated response varies for an identical perpetrator, since bosutinib and alectinib sit at opposite extremes, with the first prohibited outright and the second requiring no change at all. Two further judgments follow from the mechanistic categories in Table 3. The duration of an interaction must be matched to its mechanism, meaning that a washout is planned for induction but not for reversible inhibition, and the direction of an interaction must be established rather than assumed, because the anticancer agent may be the perpetrator.

Monitoring is tailored to the agent and to the anticipated interaction, with blood counts for the PARP inhibitors, electrocardiography for the agents that prolong the QT interval such as ribociclib and nilotinib, and liver function tests where hepatotoxicity is the dose-limiting concern [40,43,79]. Genotyping for CYP2D6 before tamoxifen and for UGT1A1 before an irinotecan-containing regimen addresses the inherited component of the same enzymatic variability, as set out in Section 7 [132,134,152,153]. These activities are most effective when they are embedded in the care pathway rather than performed ad hoc, and when they are supported by decision support that flags interactions at the point of prescribing [59,98].

## 9. The Three Consequences and What They Change

The three consequences named in the introduction are developed separately across the preceding sections. Each is set out here with the evidence that supports it, the respect in which it departs from the way interaction risk is ordinarily presented in oral oncology, and the work that would be needed to establish it more firmly. Each acquires its force only when agent-level data drawn from several sections are read against one another, which is the reason they are brought together at this point rather than asserted at the outset.

### 9.1. Interaction Liability Resides in the Molecule Rather than in the Therapeutic Class

Agents that share a therapeutic target do not share an interaction profile, and the range within a single class is wide enough to make class-level statements unusable. The three covalent inhibitors of Bruton tyrosine kinase are the clearest case, since all three engage the same active-site cysteine, and yet a strong inhibitor of CYP3A raises the exposure of ibrutinib twenty-four-fold, of acalabrutinib 5.1-fold and of zanubrutinib 3.8-fold. The four PARP inhibitors are cleared by four different routes, namely CYP3A4 and CYP3A5 for olaparib, CYP2D6 for rucaparib, carboxylesterase hydrolysis followed by glucuronidation for niraparib, and renal excretion of unchanged drug for talazoparib, so only one of the four is meaningfully exposed to cytochrome P450 modulation at all. Across the whole dataset the extremes run from a fifteen-fold rise in everolimus exposure with ketoconazole to no clinically meaningful change for alectinib with either a strong inhibitor or a strong inducer.

The contribution this makes is to move the unit of interaction risk from the class to the molecule. Guidance and formulary material commonly organize interaction warnings by therapeutic class, and a statement that kinase inhibitors are CYP3A4 substrates is at once true of the group and uninformative about any member of it. The evidence assembled here shows why the generalization fails, since the enzyme that clears a molecule is fixed by the chemistry that gave it affinity for its target rather than by the target itself, meaning that the two properties are independent at the level of the class while remaining coupled at the level of the individual agent. Establishing this more firmly requires harmonized determination of the fraction metabolized by each enzyme within therapeutic classes, because the present record is assembled from separate development programs whose studies were never designed to be compared with one another.

### 9.2. The Mechanism of Inhibition Determines the Duration of an Interaction

Magnitude and mechanism are separate properties of an interaction, and it is the mechanism that determines what happens once the perpetrator is stopped. Reversible inhibition resolves as the perpetrator is eliminated, so the interaction lasts as long as the perpetrator does. Time-dependent inhibition inactivates the enzyme, meaning that recovery waits on resynthesis, and the glucuronide of gemfibrozil that inactivates CYP2C8 is the clearest instance, since its offset is governed by enzyme turnover rather than by the elimination of the drug the prescriber withdrew [126,127]. Induction operates on a third timescale, requiring transcriptional upregulation to develop and a comparable period to decay, which is why the exposure of dabrafenib on day 15 is 47% lower than on day 1 and why it reduces midazolam exposure by 74%. The same separation holds at the therapeutic target, where covalent engagement persists after the drug itself has been cleared [116].

The contribution this makes is to attach a time course to interactions that are ordinarily recorded only by severity. An alert that reports magnitude alone carries the implicit suggestion that separating administration or waiting for the perpetrator to clear will resolve the problem, which is true of competitive inhibition and false of the other two mechanisms. Recording mechanism alongside magnitude changes the management decision, because it determines whether a washout is required at all and, when it is, how long it must last. The research this points to is the characterization of onset and offset. Registration studies are designed to establish the magnitude of an interaction at steady state, with the consequence that the time course of recovery after a mechanism-based inactivator or an inducer is withdrawn has not been measured for most of the agents used in oral oncology.

### 9.3. The Anticancer Agent May Be the Perpetrator Rather than the Victim

Interaction assessment in oncology is habitually organized around the anticancer agent as the drug at risk, and the agents collected in Table 4 show how often the reciprocal effect is the larger one. Idelalisib raises midazolam exposure 5.4-fold, which exceeds the effect of several of the antifungals ordinarily discussed as perpetrators, and it does so while being itself a therapeutic inhibitor of an entirely different enzyme. Dabrafenib and encorafenib induce the enzymes responsible for their own clearance, meaning that single-dose data overestimate steady-state exposure and a co-medication started at the beginning of treatment may become subtherapeutic without any change to its own dose. Nilotinib and rucaparib inhibit UGT1A1, asciminib inhibits CYP3A4 and CYP2C9, and vemurafenib inhibits a broad range of cytochrome P450 isoforms.

The contribution this makes is to require that the direction of an interaction be established rather than assumed. A screen run in one direction only, asking what the co-medications do to the anticancer agent, leaves the reciprocal effect undetected even when it is the larger of the two, and auto-induction is best understood as a perpetrator effect that an agent exerts upon itself, rather than as a peculiarity of its own pharmacokinetics. The research this points to is the systematic characterization of approved oral anticancer agents against probe substrates, since Table 4 is short not because the effect is uncommon but because it has been measured for comparatively few agents. Until that is done, the absence of a perpetrator entry for an agent should be read as an absence of study rather than as evidence of no effect.

## 10. Limitations

The evidence assembled here has boundaries that should be stated plainly rather than left for the reader to infer. The most important of these concerns the management sequence described in Section 8. Each of its components is individually well-founded, but prospective evidence that the integrated process improves toxicity, dose intensity or survival is absent, and the workflow should therefore be read as a defensible synthesis of labeling requirements and mechanistic reasoning rather than as an intervention validated against clinical outcomes. It is for this reason that the review presents these strategies as the application of enzymology in practice rather than as a demonstrated improvement in clinical outcome, which the current literature does not support.

The quantitative comparisons in Table 2 and Table 4 are drawn from regulatory pharmacology, which is both their strength and their limitation. Interaction studies submitted for registration are typically single-perpetrator and single-victim studies conducted in healthy volunteers or selected patient cohorts, using a strong index inhibitor such as ketoconazole or itraconazole and a strong index inducer such as rifampin. They therefore describe the maximum effect attributable to one pathway rather than the compound effect of the several moderate perpetrators that a patient with polypharmacy is more likely to be taking, and they say little about enzyme activity in hepatic impairment, in the very elderly, or in the presence of the inflammation-associated suppression of cytochrome P450 activity that advanced malignancy can produce.

The absence of evidence is distributed unevenly across agents and is not the same as evidence of absence. Rucaparib and niraparib have no dedicated study of a strong CYP3A inhibitor, and the effect of an inducer has not been established for several agents in wide clinical use. These entries have been recorded as unstudied rather than as showing no effect, but a reader comparing rows should bear in mind that an empty cell reflects what was studied at registration, which in turn reflects the judgment of the sponsor and the regulatory expectations of the time, rather than the clinical importance of the question. In vitro potency values are likewise not comparable across agents, since they are drawn from separate development programs using different assay formats and ATP concentrations. Michaelis constants for the metabolism of these agents by specific cytochrome P450 isoforms, which would permit a more rigorous quantitative treatment of competitive inhibition, are largely absent from the public record, and the fraction metabolized by a given enzyme, together with the observed clinical exposure ratio, are the practical substitutes used throughout this review. Finally, product labeling is revised over time, and all values are stated as current at a defined date.

## 11. Future Directions

Several developments would materially improve the prediction and management of enzyme-mediated interactions in oral oncology, and the gaps identified in Section 10 indicate where effort is best directed. Physiologically based pharmacokinetic modeling is increasingly used to anticipate interactions during drug development and to inform labeling, and the field continues to refine its approaches and standards [120]. Its most valuable near-term application would be precisely where clinical study data are absent, namely in predicting the compound effect of several moderate perpetrators acting on the same enzyme, and in extending single-pathway registration findings to patients with organ impairment. Population pharmacokinetic and model-informed dosing frameworks extend the same quantitative reasoning to individual patients [94,95].

Pharmacogenomically guided dosing will broaden as actionable evidence accumulates beyond the established CYP2D6 and UGT1A1 paradigms, including germline determinants of kinase-inhibitor outcomes [69,153]. Publication of a consortium guideline for UGT1A1 and irinotecan, currently identified as a priority but not yet issued, would resolve the present situation in which the actionable recommendation rests on one national working group together with product labeling. Three further priorities follow directly from the comparisons presented here. The interaction studies that were never conducted should be completed, since agents in wide clinical use without a strong-inhibitor or strong-inducer study represent avoidable uncertainty. Standardized and harmonized determinations of target potency and of the fraction metabolized within therapeutic classes would allow for the kind of quantitative cross-agent comparison that the present regulatory record cannot support. Most consequentially, the management sequence described in Section 8 should be evaluated prospectively against toxicity, dose intensity and survival, because until that is done the field will continue to recommend with confidence a process whose components are validated but whose aggregate benefit is assumed.

## 12. Conclusions

The strongest result to emerge from the agent-level data assembled here is that a shared therapeutic target predicts very little about how an agent will behave in the presence of a perpetrator. Dependence on a single metabolizing enzyme varies widely within every therapeutic class examined, and the divergence is greatest between agents that are otherwise most alike, including agents that engage the same residue of the same target by the same mechanism. No generalization made at the level of the drug class survives that spread. The cause is specific rather than incidental, since the chemistry that gives a molecule affinity for its target is the same chemistry that determines which enzyme clears it, meaning that potency and interaction liability are generated by one set of structural decisions rather than by two. The contribution of this review is to establish that relationship quantitatively across seven target classes and to draw from it the perspective that interaction risk belongs to the molecule and not to its class.

Two things follow on from this. Regarding practice, the mechanism of inhibition must be recorded alongside its magnitude, because magnitude states how large an interaction is while mechanism states how long it lasts and whether withdrawing the perpetrator will end it, and the questions asked at the point of prescribing become determinate: which enzyme dominates clearance and how completely, by what mechanism that enzyme can be perturbed, in which direction the perturbation runs given that an anticancer agent may inhibit or induce as readily as it is inhibited or induced, and what the labeling requires when it does. The choice between two agents of equivalent efficacy is thereby a pharmacological decision, since for a patient established on long-term azole prophylaxis they are not equally safe. Regarding research, three studies would settle what a review of the present literature can only suggest. Harmonized determination of the fraction metabolized by each enzyme within therapeutic classes, under common assay conditions, would make cross-agent comparison quantitative rather than indicative. Characterization of the onset and offset of interactions, which registration studies are not designed to provide, would convert the mechanistic classification into prescribable washout intervals. Prospective evaluation of the management sequence against toxicity, dose intensity and survival would establish whether reasoning of this kind changes outcomes, which at present is assumed rather than demonstrated.

## Figures and Tables

**Table 1:** Therapeutic enzyme targets in oral anticancer therapy and the enzymological issue governing the use of each class.

Enzyme Target	Representative Oral Inhibitors	Mechanism of Inhibition	Main Indication	Enzymological Issue Governing Use
BCR-ABL1	Imatinib, dasatinib, nilotinib, bosutinib, ponatinib, asciminib	ATP-competitive, with asciminib allosteric at the myristoyl pocket	Chronic myeloid leukemia	Kinase-domain mutations, including T315I, and a twenty-fold range of CYP3A4 dependence across the class (Table 2) [26,27,28]
EGFR, ALK and MET	Erlotinib, gefitinib, afatinib, osimertinib, crizotinib, ceritinib, alectinib, lorlatinib	ATP-competitive, with afatinib and osimertinib covalent	Non-small cell lung cancer	Resistance mutations, and clearance routes ranging from CYP3A dominance to negligible enzymatic metabolism (Table 2) [29,30,31]
VEGFR	Lenvatinib, cabozantinib, axitinib, sorafenib, regorafenib, pazopanib	ATP-competitive, multikinase	Renal, thyroid and hepatic tumors	Clearance divided between CYP3A4 and UGT1A9, so glucuronidation can buffer the loss of oxidative clearance [32,33,34]
BTK	Ibrutinib, acalabrutinib, zanubrutinib	Covalent, at an active-site cysteine	B-cell malignancies	Off-target kinase inhibition explains the toxicity split, and first-pass extraction explains a six-fold range of interaction magnitude (Table 2) [35,36,37]
PARP1 and PARP2	Olaparib, rucaparib, niraparib, talazoparib	Catalytic inhibition together with PARP trapping	Homologous-recombination-deficient tumors	Four agents cleared by four different mechanisms, only one of which is CYP3A-dependent (Table 2) [38,39,40]
CDK4 and CDK6	Palbociclib, ribociclib, abemaciclib	ATP-competitive	HR-positive, HER2-negative breast cancer	All three are CYP3A4 substrates, but induction rather than inhibition is the dominant hazard (Table 2) [41,42,43]
PI3K, AKT and mTOR	Idelalisib, everolimus, sirolimus	ATP-competitive, with the mTOR agents allosteric via FKBP-12	Lymphoma, breast and renal tumors	The mTOR agents are the most CYP3A4-sensitive drugs considered here, and idelalisib is itself a strong CYP3A inhibitor [44,45,46]
BRAF and MEK	Vemurafenib, dabrafenib, encorafenib, trametinib, cobimetinib	ATP-competitive at BRAF, allosteric at MEK	BRAF-mutant melanoma	Paradoxical MAPK activation, and auto-induction of clearance by dabrafenib and encorafenib (Section 6) [47,48,49]
HDAC	Vorinostat, panobinostat	Zinc-binding catalytic inhibition	Myeloma and T-cell lymphoma	Clearance route rather than class determines interaction risk, since vorinostat is non-CYP and panobinostat is CYP3A-dependent [50,51,52]
Proteasome (20S)	Ixazomib	Catalytic-site binding at the beta-5 subunit	Multiple myeloma	No single enzyme dominates clearance, so induction rather than inhibition is the actionable concern [53,54,55]

Cediranib and veliparib appear in the earlier literature but have never received marketing authorization and are therefore excluded. Belinostat and romidepsin are administered intravenously and are discussed only as mechanistic comparators in Section 5.

**Table 2:** Target-enzyme inhibition and metabolizing-enzyme dependence for representative oral anticancer agents, grouped by therapeutic class.

Agent (Target Class)	Target Potency, IC50 or Ki (Assay Type)	Approved Adult Oral Dose	Mechanism of Target Inhibition	Enzyme Dominating Clearance	Strong Inhibitor of That Enzyme: Perpetrator and Change in AUC	Rifampin: Change in AUC	Dose Modification Mandated by Labeling
Ibrutinib (BTK)	BTK 0.39–0.5 nM (purified enzyme). EGFR 5.3, ITK 4.9, TEC 10 nM	420 mg once daily	Covalent, active-site cysteine	CYP3A	Ketoconazole, 24-fold increase. Voriconazole, 5.7-fold	Decrease exceeding 10-fold	Posaconazole 70 mg daily, voriconazole 140 mg daily, otherwise avoid
Acalabrutinib (BTK)	BTK 3.0–5.1 nM. EGFR and ITK both above 1000 nM	100 mg every 12 h	Covalent, active-site cysteine	CYP3A	Itraconazole, 5.1-fold increase	Decrease of 77%	Avoid strong inhibitors; reduce to 100 mg once daily with a moderate inhibitor
Zanubrutinib (BTK)	BTK 1.8 nM (cellular), 0.3 nM (cell-free)	160 mg twice daily or 320 mg once daily	Covalent, active-site cysteine	CYP3A	Itraconazole, 3.8-fold increase	Decrease of 93%	Reduce to 80 mg once daily with a strong inhibitor
Olaparib (PARP)	PARP1 5 nM, PARP2 1 nM	300 mg twice daily	Catalytic inhibition and trapping	CYP3A4 and CYP3A5	Itraconazole, increase of 170%	Decrease of 87%	Reduce to 100 mg twice daily with a strong inhibitor
Rucaparib (PARP)	PARP1 Ki 1.4 nM, PARP2 Ki 0.17 nM	600 mg twice daily	Catalytic inhibition and trapping	CYP2D6, with CYP1A2 and CYP3A4 minor	No dedicated clinical CYP3A study exists	Not studied	None specified
Niraparib (PARP)	PARP1 1.1–2.8 nM, PARP2 0.4–0.6 nM	200–300 mg once daily by weight and platelet count	Catalytic inhibition and trapping	Carboxylesterase hydrolysis, then glucuronidation	No dedicated clinical CYP3A study exists	Not studied	None specified
Talazoparib (PARP)	PARP1 0.7 nM, PARP2 0.3 nM. Strongest trapping of the class	1 mg once daily	Catalytic inhibition and trapping	Minimal metabolism; renal excretion of unchanged drug	Not an enzyme interaction. Itraconazole increases AUC 56% by inhibiting P-glycoprotein	No change in AUC	Reduce to 0.75 mg daily with named P-glycoprotein inhibitors
Imatinib (BCR-ABL1)	Cellular IC50 260 nM (Ba/F3)	400 mg once daily (chronic phase)	ATP-competitive, reversible	CYP3A4 major, CYP2C8 minor	Ketoconazole, increase of 40%	Decrease of at least 74%	No numeric reduction; caution advised
Bosutinib (BCR-ABL1)	Abl 1 nM, Src 1.2 nM (cell-free). Inactive against T315I	400–500 mg once daily	ATP-competitive, reversible	CYP3A4	Ketoconazole, 8.6-fold increase	Decrease of 94%	Avoid strong and moderate inhibitors entirely
Asciminib (BCR-ABL1)	Wild-type 0.61 nM, T315I 7.64 nM (kinase assay)	80 mg once daily; 200 mg twice daily for T315I	Allosteric, myristoyl pocket	CYP3A4 with UGT2B7 and UGT2B17	Clarithromycin, increase of 36%	Decrease of 15%	No reduction specified; monitor at the 200 mg twice-daily dose
Palbociclib (CDK4/6)	CDK4 11 nM, CDK6 15 nM	125 mg once daily, 21 of 28 days	ATP-competitive, reversible	CYP3A and SULT2A1	Itraconazole, increase of 87%	Decrease of 85%	Reduce to 75 mg daily with a strong inhibitor
Ribociclib (CDK4/6)	CDK4 8 nM, CDK6 39 nM	400–600 mg once daily, 21 of 28 days	ATP-competitive, reversible	CYP3A4	Ritonavir, 3.2-fold increase	Decrease of 89%	Reduce 600 mg to 400 mg daily with a strong inhibitor
Abemaciclib (CDK4/6)	CDK4 2.0 nM, CDK6 1.6 nM	150 mg twice daily, continuous	ATP-competitive, reversible	CYP3A4, with three active metabolites	Clarithromycin, 2.5-fold increase in potency-adjusted unbound exposure	Decrease of approximately 70%	Reduce to 100 mg twice daily; avoid ketoconazole
Osimertinib (EGFR)	T790M/L858R 1 nM, wild-type 184 nM (recombinant enzyme)	80 mg once daily	Covalent, mutant-selective	CYP3A	Itraconazole, increase of 24% and therefore a negative study	Decrease of 78%	None required for inhibitors; increase to 160 mg with an inducer
Ceritinib (ALK)	ALK 0.15 nM (biochemical), 27–35 nM (cellular)	450 mg once daily	ATP-competitive, reversible	CYP3A	Ketoconazole, 2.9-fold increase	Decrease of 70%	Reduce dose by approximately one third
Alectinib (ALK)	ALK 1.9 nM, metabolite M4 1.2 nM (enzyme assay)	600 mg twice daily	ATP-competitive, reversible	CYP3A4, to the active metabolite M4	Posaconazole, no clinically meaningful effect	No clinically meaningful effect	None required
Dabrafenib (BRAF)	V600E 0.65 nM, wild-type BRAF 3.2 nM	150 mg twice daily	ATP-competitive, reversible	CYP2C8 and CYP3A4	Ketoconazole, increase of 71%. Gemfibrozil, increase of 47%	Decrease of 34%	Avoid strong CYP3A4 or CYP2C8 inhibitors
Trametinib (MEK)	MEK1 activation 0.7 nM, MEK2 0.9 nM	2 mg once daily	Allosteric, outside the ATP site	Carboxylesterases, not cytochrome P450	No study, consistent with non-CYP clearance	Not studied	None
Everolimus (mTOR)	Acts via the FKBP-12 complex; no numeric value reported	10 mg once daily (oncology)	Allosteric	CYP3A4	Ketoconazole, 15-fold increase. Erythromycin, 4.4-fold	Decrease of 63%	Avoid strong inhibitors; reduce to 2.5 mg daily with a moderate inhibitor
Vorinostat (HDAC)	HDAC1, 2, 3 and 6 below 86 nM	400 mg once daily	Zinc-binding catalytic inhibition	Glucuronidation and hydrolysis; cytochrome P450 negligible	No data; not a cytochrome P450 substrate	Not studied	None
Ixazomib (proteasome)	Beta-5 subunit 3.4 nM, beta-1 31 nM	4 mg once weekly, days 1, 8 and 15 of 28	Catalytic-site binding	No single dominant enzyme	Clarithromycin, no clinically meaningful change	Decrease of 74%	None for inhibitors; avoid strong inducers

Values were taken from United States prescribing information, European Medicines Agency assessment reports and Summaries of Product Characteristics, or FDA review documents, current on 6 September 2026. Potency values derive from separate development programs using non-harmonized assays and characterize each agent rather than permitting a ranking across agents. An entry recording that no study exists denotes an absence of data and not a demonstration of no effect.

**Table 3:** Mechanistic categories of enzyme inhibition and induction relevant to oral anticancer therapy, spanning both the therapeutic target and the metabolizing enzyme.

Mechanism	Molecular Basis	Oncology Example	Consequence for Management
ATP-competitive inhibition (therapeutic)	Competes with ATP at the catalytic cleft, so the effect tracks the prevailing plasma concentration	Most kinase inhibitors	Pharmacodynamic effect follows exposure, which is why exposure-altering interactions translate directly into altered efficacy [1,6,56]
Allosteric inhibition (therapeutic)	Binds a site distinct from the catalytic center	Asciminib at the myristoyl pocket, the MEK inhibitors, the mTOR inhibitors via FKBP-12	Can bypass resistance mutations that arise at the ATP site [27,49]
Covalent inhibition (therapeutic)	Forms a bond with an active-site residue, usually a cysteine	BTK inhibitors, afatinib, osimertinib	Target engagement persists after the drug has cleared, so effect and exposure are uncoupled [35,116]
Reversible metabolic inhibition	Competitive inhibition of a metabolizing enzyme by a co-administered drug	Azole antifungals inhibiting CYP3A4	The effect reverses as the perpetrator is eliminated, and its magnitude scales with the victim’s dependence on that enzyme [5,9]
Time-dependent inhibition	Catalytic inactivation of the enzyme, often through a reactive metabolite, or through a phase II conjugate of the perpetrator rather than the parent drug	Metabolic activation of certain tyrosine kinase inhibitors, and gemfibrozil glucuronide acting on CYP2C8	The effect is longer lasting and potentially cumulative, and persists until enzyme is resynthesized [1,75]
Enzyme induction	Transcriptional upregulation of enzyme expression, which lowers substrate exposure	Rifampin, the enzyme-inducing antiepileptics, enzalutamide	Onset and offset are slow, so a washout is required before a sensitive substrate is started [10,142]
Auto-induction	The agent induces the enzymes responsible for its own clearance	Dabrafenib and encorafenib	Steady-state exposure is lower than single-dose data predict, and co-medication exposure falls in parallel (Section 6) [48]

## Data Availability

No new data were created or analyzed in this study. All values reported in Table 2 and Table 4 were extracted from publicly available regulatory documents, namely United States prescribing information, European Medicines Agency assessment reports and Summaries of Product Characteristics, and FDA review documents, each of which is cited in the reference list. All other data referenced are available in the cited publications.

## Abbreviations

The following abbreviations are used in this manuscript: ABCB1, ATP-binding cassette subfamily B member 1; ABCG2, ATP-binding cassette subfamily G member 2; ALK, anaplastic lymphoma kinase; AUC, area under the concentration–time curve; BCRP, breast cancer resistance protein; BTK, Bruton tyrosine kinase; CDK, cyclin-dependent kinase; Cmax, maximum plasma concentration; CYP, cytochrome P450; EGFR, epidermal growth factor receptor; EMA, European Medicines Agency; FDA, United States Food and Drug Administration; FKBP-12, FK506-binding protein 12; HDAC, histone deacetylase; HER2, human epidermal growth factor receptor 2; HR, hormone receptor; IC50, half-maximal inhibitory concentration; Ki, inhibition constant; Km, Michaelis constant; MAPK, mitogen-activated protein kinase; MEK, mitogen-activated protein kinase kinase; MET, mesenchymal–epithelial transition factor; mTOR, mechanistic target of rapamycin; PARP, poly(ADP-ribose) polymerase; PI3K, phosphatidylinositol 3-kinase; P-gp, P-glycoprotein; PXR, pregnane X receptor; QT, QT interval of the electrocardiogram; SN-38, 7-ethyl-10-hydroxycamptothecin; TKI, tyrosine kinase inhibitor; UGT, uridine diphosphate glucuronosyltransferase; VEGFR, vascular endothelial growth factor receptor.
